# Processing of the same narrative stimuli elicits common functional connectivity dynamics between individuals

**DOI:** 10.1038/s41598-023-48656-7

**Published:** 2023-12-02

**Authors:** Başak Türker, Laouen Belloli, Adrian M. Owen, Lorina Naci, Jacobo D. Sitt

**Affiliations:** 1 Institut du Cerveau - Paris Brain Institute - ICM, Inserm, CNRS, Sorbonne Université, 75013 Paris, France; 2 Instituto de Ciencias de la Computacion, CONICET-UBA, Buenos Aires, Argentina; 3 The Western Institute for Neuroscience, Western Interdisciplinary Research Building, University of Western Ontario, London, ON N6A 5B7 Canada; 4 Trinity College Institute of Neuroscience, School of Psychology, Trinity College Dublin, Lloyd Building, Dublin, Ireland

**Keywords:** Consciousness, Perception

## Abstract

It has been suggested that conscious experience is linked to the richness of brain state repertories, which change in response to environmental and internal stimuli. High-level sensory stimulation has been shown to alter local brain activity and induce neural synchrony across participants. However, the dynamic interplay of cognitive processes underlying moment-to-moment information processing remains poorly understood. Using naturalistic movies as an ecological laboratory model of the real world, here we investigate how the processing of complex naturalistic stimuli alters the dynamics of brain network interactions and how these in turn support information processing. Participants underwent fMRI recordings during movie watching, scrambled movie watching, and resting. By measuring the phase-synchrony between different brain networks, we analyzed whole-brain connectivity patterns. Our finding revealed distinct connectivity patterns associated with each experimental condition. We found higher synchronization of brain patterns across participants during movie watching compared to rest and scrambled movie conditions. Furthermore, synchronization levels increased during the most engaging parts of the movie. The synchronization dynamics among participants were associated with suspense; scenes with higher levels of suspense induced greater synchronization. These results suggest that processing the same high-level information elicits common neural dynamics across individuals, and that whole-brain functional connectivity tracks variations in processed information and subjective experience.

## Introduction

The content of our conscious experience changes depending on the environment and ongoing task. Both external and internal information are processed and integrated to give rise to our conscious experiences. The dynamic interplay of cognitive processes that underlie our moment-to-moment experience of the world remains poorly understood. Using naturalistic movies as an ecological laboratory model of the real world, previous studies have shown that audio-visual clips influence brain activity in a similar manner across individuals, reflecting their shared conscious experiences and holistic understanding. Movie watching can synchronize brain activity in the cortex^1–6^, elicit time-resolved correlations between pairs of regions^7^, and give rise to consistent whole-brain activations across participants^8^. Moreover, studies have shown that the quality of encoding of the movie’s content is correlated with inter-subject synchronization during movie watching^9,10^. However, the descriptions of local activations offer only a limited summary of the dynamic processes that give rise to coherent understanding over time.

In recent years, dynamic descriptions of brain activity have gained prominence as they might better account for the participant’s mental state. Focusing on how different brain networks interact over time, rather than the classic description of local activity, could provide a better understanding of conscious processing. The interaction between brain regions has been widely investigated using static functional connectivity^11–14^ computed over the entire fMRI scan (for a comprehensive review see^15^). More recently, dynamic functional connectivity measures came into use, revealing transient brain states that vary in time^16–20^, reflecting cognitive processes at any given moment^21,22^. It has been suggested that the richness of conscious experience can be directly linked to the richness of brain state repertories. Indeed, individuals who lack consciousness present brain states that are less diverse, with fewer long-range interactions and no anticorrelation between brain areas^23–25^. Furthermore, active interventions, such as deep brain stimulation aimed at restoring consciousness have been found to increase the diversity of brain state repertoires^26^.

A recent study utilizing a latent space representation of brain network interactions found increased overall similarity between participants who actively tried to understand a scrambled movie^27^. However, the movie features that drive inter-subject synchronization of brains states are poorly understood. Given the evolving nature of movie plots, high-level cognitive processing of the movie varies significantly over time. Therefore, it is crucial to unravel the relationship between similar brain state dynamics across participants and the dynamic features of a particular narrative to understand how brain processes contribute to movie comprehension over time.

To address this gap, our study investigates how the processing of plot-driven naturalistic movies dynamically shapes brain state repertoire, as assessed through interactions among dynamic brain networks. This brain state repertoire has previously been shown to undergo alterations in unconscious states^25^. In our study, we examine whole-brain connectivity patterns that emerge during resting-state, movie watching, and scrambled movie watching in healthy participants to assess the functional roles of these brain states and how they evolve during shared conscious experiences. Our findings demonstrate that certain brain patterns are more prevalent during movie-watching whereas some others are more prominent in non-movie conditions. Moreover, by analyzing temporal average and dynamic (time-resolved) synchronization of the connectivity patterns between participants, we show that narrative stimuli induce higher inter-subject synchronization, especially during suspenseful scenes. Altogether, these results suggest that processing of the same narrative stimuli elicits common functional connectivity configurations between individuals, and the dynamics of these brain states track variations in the high-level properties of the processed information.

## Results

**Figure 1:**
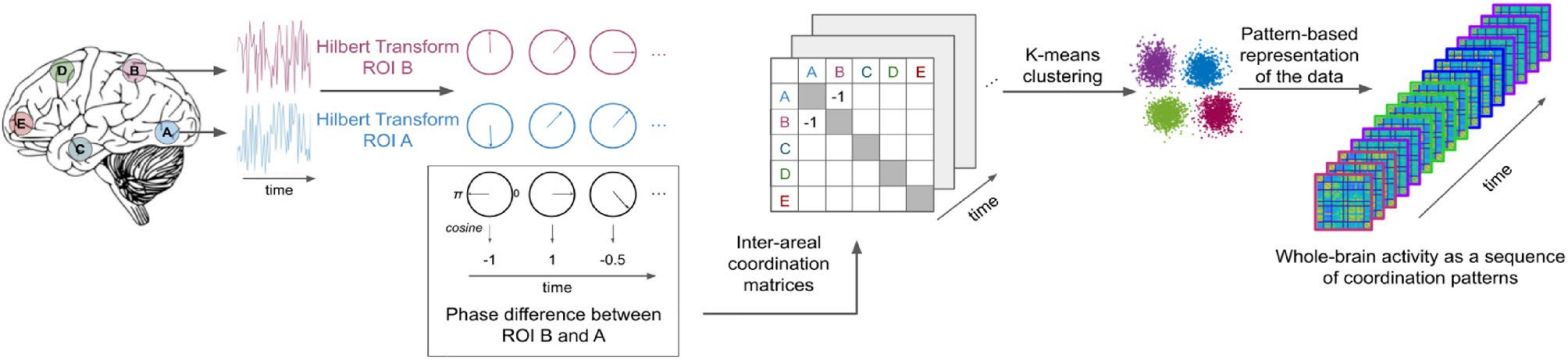
Summary of the method used to compute inter-areal connectivity patterns. Following the preprocessing of the fMRI data, BOLD signal time-series were extracted from 42 regions of interest (ROIs) belonging to 6 different networks (visual, auditory, saliency, default mode, fronto-parietal, and motor). The Hilbert transform was applied to all time-series in order to extract the instantaneous phase at each fMRI volume (pink and blue circles in the figure). Phase-differences were computed between each ROI pair (in this example between ROI A and B) at each time point (black circles in the figure). Using cosine similarity, we ranged the phase synchronies between − 1 and 1; − 1 indicating a complete phase opposition and 1 indicating a complete phase coherence between the two ROIs. Then, phase synchrony values were used to create a 42 by 42 inter-areal coordination matrix for each time-point (fMRI volume). Using k-means clustering, we classified the coordination matrices into 4 *prototypical patterns*. Finally, the fMRI data of each participant was expressed as a sequence of these 4 patterns.

**Figure 2:**
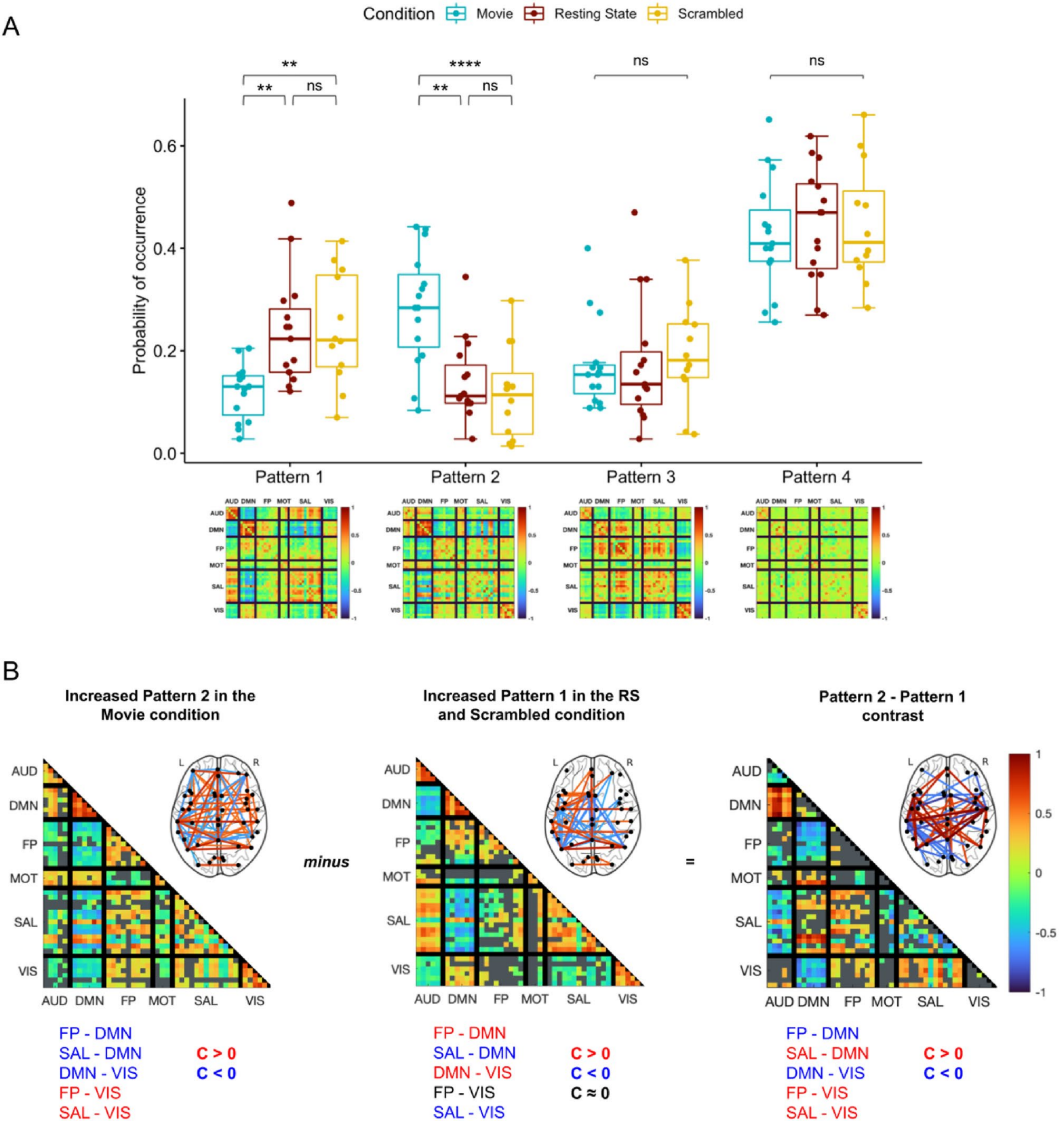
Condition specific variation of the whole-brain connectivity patterns. (**A**) Occurrence probability of the 4 patterns during movie watching (blue), rest (brown) and scrambled movie watching (yellow). Patterns 3 and 4 displayed similar occurrence probabilities across the three conditions. However, Patterns 1 and 2 showed condition-specific modulation: while Pattern 2 showed increased occurrence during movie watching, Pattern 1 was more frequent in scrambled movie watching and resting-state conditions. Stars indicate statistical significance after correction for multiple comparisons using the Bonferroni procedure. (**B**) Description of the differences between Pattern 2 (more frequent in movie condition) and Pattern 1 (more frequent in non-movie conditions). Coherence values are depicted using blue shades for negative coherence (C < 0) and red shades for positive coherence (C > 0) after correction for multiple comparisons using the Benjamini–Hochberg procedure (Sign test; *p* < 0.001). Gray color represents coherence that is not significantly different from zero. Note that brain templates show top 5% coherence for visualisation purposes. Pattern 2 (left), which was more frequent in the movie condition, showed negative phase coherence between the default-mode network (DMN) and fronto-parietal (FP), saliency (SAL) and visual (VIS) networks while the visual network had positive coherence with fronto-parietal (FP) and saliency (SAL) networks. Pattern 1 (center), which had more occurrence in non-movie conditions, was characterized by positive coherence between default-mode network (DMN) and fronto-parietal (FP) and visual (VIS) networks and negative coherence between saliency network (SAL) and visual (VIS) and default-mode networks. The contrast between patterns (Pattern 2–Pattern 1; right) revealed more negative coherence between default-mode network (DMN) and fronto-parietal (FP) and visual (VIS) networks in Pattern 2 (increased with movie) than Pattern 1 (increased with non-movie conditions). Moreover, saliency (SAL) and visual (VIS) networks showed more positive coherence in Pattern 2 compared to Pattern 1. Gray color indicates p values greater than 0.001 after FDR correction in a mixed linear model.

We investigated how high-level sensory information processing influences ongoing brain activity emerging from the coordination of different brain regions. 15 participants underwent fMRI recordings during movie watching and rest. A second group of 12 participants watched the same movie but scrambled to prevent them from understanding the plot while still viewing every scene. Using Hilbert transform and k-means clustering, we computed whole-brain connectivity patterns for each fMRI volume in each condition (Fig. 1). The clustering procedure included 42 regions of interests (Table S1) taken from a previous study^25^ and has resulted in four distinct connectivity patterns (Fig. 2A).

First, we hypothesized that some whole-brain connectivity patterns would be associated with a particular experimental condition and would vary in frequency accordingly. A linear model revealed a Pattern*Condition interaction (*F*(6) = 7.17, *p* < 0.0001). Pattern 2 showed increased occurrence during movie watching (median: 28.4%) compared to resting-state (median: 11.2%, *t*(156) = 3.94, *p* = 0.0004, after Bonferroni correction), and scrambled movie watching (median: 11.4%, *t*(156) = 4.77, *p* < 0.0001). By contrast, Pattern 1 was less frequent during movie watching (median: 13.0%) compared to resting-state (median: 22.3%, *t*(156) = − 3.48, *p* = 0.0019) and scrambled movie (22.1%, *t*(156) = − 3.38, *p* = 0.0027) (Fig. 2A). We didn’t find any significant difference between the resting-state and scrambled movie watching for Patterns 1 (*t*(156) = − 0.099, *p* = 1.00) and Pattern 2 (*t*(156) = 1.05, *p* = 0.88). Patterns 3 and 4 had similar occurrence probabilities across all conditions.

To ensure that these results were not dependent on our ROI selection, we conducted a second clustering procedure using the Harvard–Oxford whole-brain parcellation atlas. The connectivity patterns found in this procedure can be found in Fig. S1. As anticipated, the use of this alternative whole-brain atlas produced similar findings. We found a Pattern*Condition interaction *F*(6) = 5.54, *p* < 0.0001). Patterns 3 and 4 did not exhibit significant differences between conditions, whereas Patterns 1 and 2 showed condition-specific changes (see Fig. S2A). Specifically, Pattern 2 had a higher occurrence during movie watching compared to both resting-state (*t(*156) = 2.46, *p* = 0.045, after Bonferroni correction), and scrambled movie watching (*t*(156) = 2.56, *p* < 0.035). Conversely, Pattern 1 was less frequent during movie watching compared to scrambled movie watching (*t*(156) = − 3.84, *p* < 0.0005). The difference between movie watching and resting state for Pattern 1 remained a tendency following strict Bonferroni correction (*t*(156) = − 2.37, *p* < 0.057). Nonetheless, the observed effect's directionality aligned with our results from the original analysis.

Given the movie-specific modulation of Patterns 1 and 2, we investigated how they differed in their inter-ROI coherence profiles. Pattern 2, which occurred more frequently during movie watching, showed positive coherence between the saliency network (SAL), fronto-parietal (FP) and visual (VIS) networks, and negative coherence between the default-mode network (DMN) and fronto-parietal (FP), saliency (SAL) and visual (VIS) networks (Fig. 2B, left panel). On the other hand, Pattern 1, which occurred more frequently in the non-movie conditions, exhibited negative coherence between SAL–DMN and SAL–VIS, and positive coherence between DMN–FP and DMN–VIS (Fig. 2B, middle panel). The contrast between Pattern 2 and Pattern 1 (Fig. 2B, right panel) revealed that Pattern 2 contained more negative coherence between DMN–FP and DMN–VIS, and more positive coherence between FP–VIS and SAL–VIS compared to Pattern 1.

**Figure 3:**
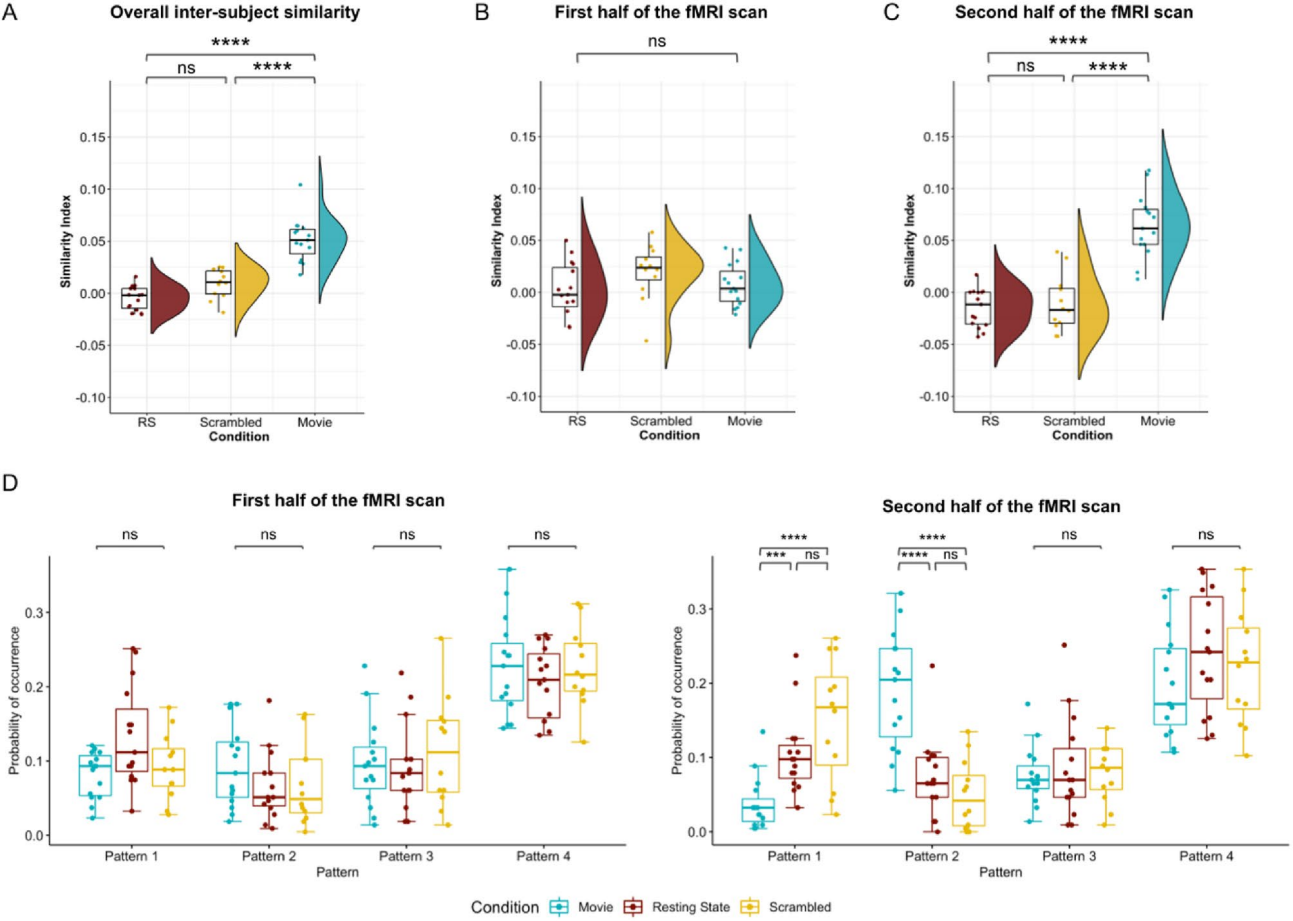
Movie watching induces inter-subject synchronization (**A**) Average inter-subject similarity index (ISI) of each participant in resting-state (RS), scrambled movie, and movie watching conditions. Similarity indices were not different than zero in non-movie conditions, indicating a chance-level co-occurrence of patterns among participants throughout the whole duration. On the other hand, participants in the movie condition showed higher similarity index values, indicating increased co-occurrence of the patterns compared to chance. Each dot represents a participant. Stars denote statistical significance after correction for multiple comparisons using Bonferroni procedure. (**B**,**C**) Average ISI of each participant computed over the first (**B**) and second (**C**) half of the fMRI scan during resting-state, scrambled movie watching, and movie watching conditions. While ISI did not differ between condition during the first half of the fMRI scan (*F*(2) = 1.73, *p* = 0.19), ISI during movie watching was significantly higher compared to resting-state (*t*(39) = 8.49, *p* < 0.0001) and scrambled movie (*t*(39) = 7.6, *p* < 0.0001) conditions. ISI during resting-state and scrambled movie conditions were not statistically different (*t*(39) = 0.41, *p* = 1). (**D**) The occurrence probability of the patterns during the first (left) and the second (right) half of the fMRI recording in movie watching, resting-state and scrambled movie watching conditions. While no significant interactions were found between the condition and the pattern probabilities during the first half of the scan (*F*(6) = 1.81, *p* = 0.1), a significant interaction was observed in the second half (*F*(2) = 13.00, *p* < 0.0001). Pattern 2 showed increased probability during movie watching compared to resting-state (*t*(156) = 5.09, *p* < 0.0001) and scrambled movie watching (*t*(156) = 6.43, *p* < 0.0001) and Pattern 1 was more frequent during scrambled movie watching (*t*(156) = 5.20, *p* < 0.0001) and resting-state (*t*(156) = 3.82, *p* = 0.0006) compared to movie watching. Patterns 3 and 4 did not differ between conditions.

Next, we hypothesized that functional connectivity dynamics would be similar across participants during movie watching when the same narrative drives brain activity. We computed an inter-subject similarity index (ISI) that allows us to assess the inter-subject co-occurrence of the patterns in the three experimental conditions. Since we were describing the brain activity with only four patterns, co-occurrence of patterns could arise by chance, even though participants were under different conditions and their brain activity was completely independent. The ISI indicates how much more co-occurrence participants have compared to chance-level co-occurrence. As expected, we found ISI values around zero during rest (Wilcoxon signed rank test after Bonferroni correction: *V* = 35, *p* = 0.51) and scrambled movie watching (*V* = 64, *p* = 0.16), indicating that the co-occurrence of patterns during those conditions was at chance level. We observed a significant increase in the ISI during movie watching (median ISI = 0.05, S.E. = 0.005, *V* = 120, *p* = 0.0002) compared to rest (median ISI = 0, S.E. = 0.003, *t*(39) = 9.56, *p* < 0.0001, after Bonferroni correction) and scrambled movie watching (median ISI = 0.01, S.E. = 0.004, *t*(39) = 6.74, *p* < 0.0001), indicating higher co-occurrence when participants watched the movie (Fig. 3A). We further validated these results with our second clustering procedure using the Harvard–Oxford atlas (Figure S2B). Consistently, the ISI values exceeded chance-level ISI only during the movie-watching condition (*V* = 100. *p* = 0.021). In contrast, during both resting-state (*V* = 33, *p* = 0.14) and scrambled movie watching (*V* = 57, *p* = 0.18), the ISI values remained around zero.

We predicted that the co-occurrence of the patterns would be more important during the most engaging parts of the movie, during which the movie plot captures the viewers’ attention. We took advantage of the suspenseful nature of the movie and utilized a pre-existing dataset where a third group of 15 participants had rated the suspense of the scenes on an 8-item scale outside of the scanner^1^. We noted higher suspense values in the second half of the movie compared to the first half (mean: 1.3 vs. 0.9, Wilcoxon rank-sum rest: *z* = 2.43, *p* = 0.01). As predicted, ISI values were higher only in the second half of the movie (*F*(2) = 44.34, *p* < 0.0001) and did not differ between conditions during the first half of the recordings (*F*(2) = 1.73, *p* = 0.2) (Fig. 3B,C). The same was true for the patterns’ occurrence probabilities in the three conditions, which only differed in the second half of the recordings (Fig. 3D).

**Figure 4:**
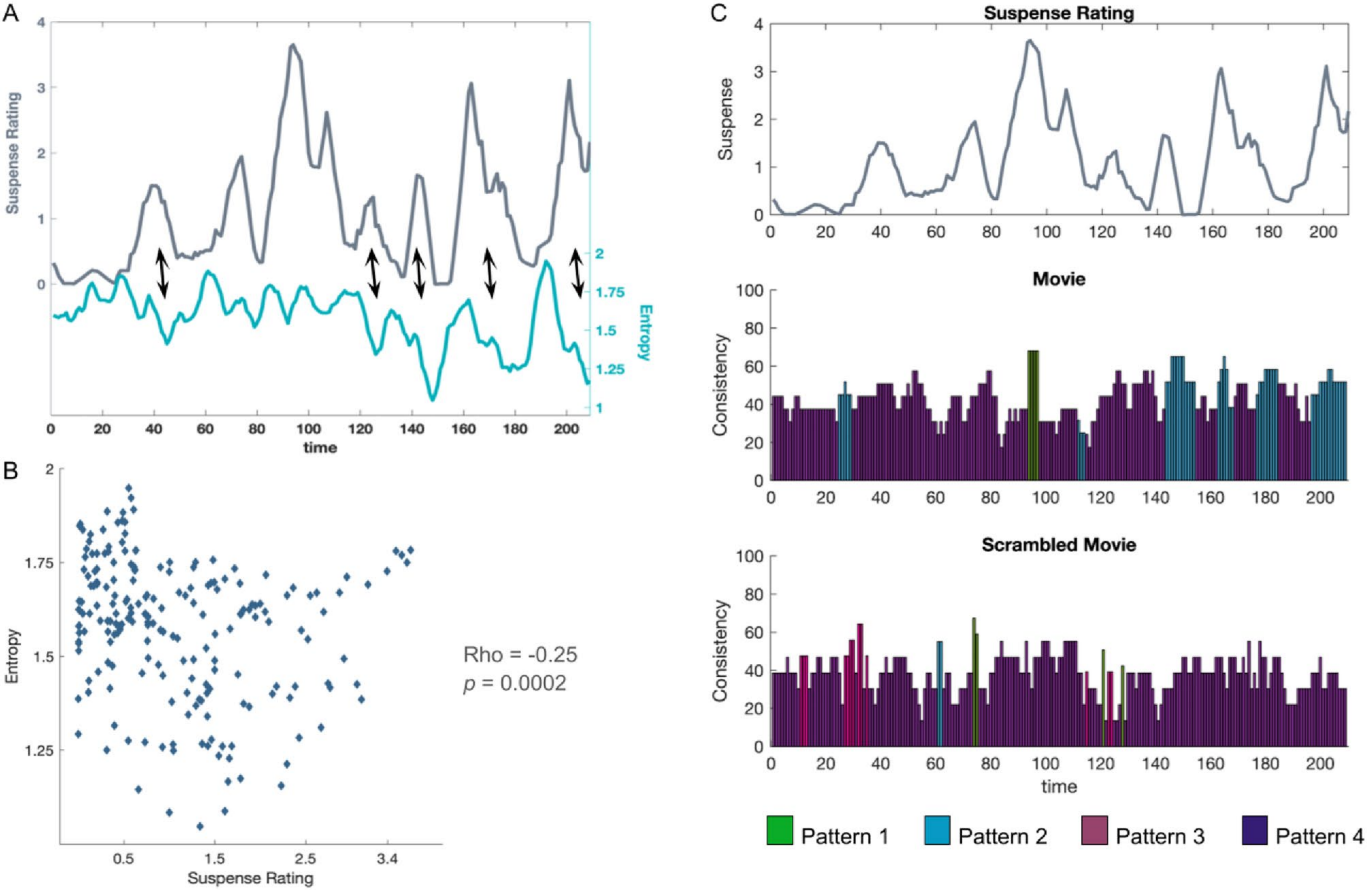
Scenes with high suspense induce inter-subject synchronization by increasing the co-occurrence of the movie-related whole-brain connectivity pattern. (**A**) Suspenseful scenes increase pattern co-occurrence during movie watching. The dark gray line shows the variations in the average suspense rating. Entropy (light blue line) of the pattern distribution among participants at each time point is used as an instantaneous co-occurrence measure. Lower entropy values indicate higher co-occurrence. Note the negative relationship between the suspense rating and the entropy values: scenes with higher suspense ratings are followed by a decrease in entropy and thus an increased co-occurrence of the patterns among participants. (**B**) Scatter plot of the average suspense rating and the subsequent entropy values (6 s after the suspense rating). We found a significant negative correlation between the two measures (*rho* = − 0.2548; *p* = 0.0002). (**C**) Instantaneous co-occurrence during suspenseful scenes is associated with the movie-specific pattern. Temporal consistency of the whole-brain connectivity patterns in the movie (middle panel) and scrambled movie (bottom panel) watching condition. Variations in the average suspense rating can be found in the top panel. The dominant pattern at a given time point is indicated by a color code. The y-axis shows the percentage of across-participant consistency. The overall occurrence probability of each pattern in the conditions (Fig. 2A) is subtracted from the instantaneous consistency in order to assess consistency exceeding random occurrence. Note the higher consistency values in the movie condition compared to the scrambled movie condition. Pattern 2 (blue) was the dominant pattern during increased suspense, reaching up to 65% excess consistency during movie watching.

For a finer assessment of inter-subject brain state synchronization, we used entropy as a measure of instantaneous pattern co-occurrence among participants at each time point. Lower entropy values indicate higher co-occurrence between participants. We observed a negative relationship between the average suspense ratings and the entropy values: scenes with higher suspense were followed by a decrease in entropy, indicating a higher co-occurrence across participants (Fig. 4A). The relationship between the suspense and entropy was further confirmed by a significant Spearman’s correlation (*rho* = − 0.2548, *p* = 0.0002), taking a 6 s fMRI response lag (3 TRs) relative to the time of suspense rating into account (Fig. 4B). This relationship was not found in the resting-state (*rho* = − 0.08, *p* = 0.25) and scrambled movie conditions (*rho* = 0.09, *p* = 0.21) (Fig. S3). The negative relationship between suspense ratings and the entropy levels during movie watching was also observed when clustering the brain patterns using the Harvard–Oxford atlas (Fig. S2C,D).

Finally, we investigated whether this increased co-occurrence during suspenseful scenes was driven by a specific connectivity pattern, or if all patterns contributed similarly to the increase. Following a previously published method^8^, we determined the most frequently expressed pattern at each time window during movie watching and scrambled movie conditions and assessed their consistency across participants (Fig. 4C). We subtracted the *time-averaged* occurrence probabilities of each pattern during the movie and scrambled movie watching (Fig. 2A) from the consistency level in these conditions to reveal consistency exceeding mean session occurrence. Overall, we found higher consistency values during movie watching compared to scrambled movie watching (mean: 44.1 vs. 38.6, Wilcoxon rank-sum test: *z* = 3.65, *p* = 0.0003). Furthermore, Pattern 2, which showed increased occurrence during movie watching, was the dominant pattern during the moments of suspense in the second half of the movie, reaching up to 65% excess consistency.

To summarize, our findings demonstrate that (1) dynamic connectivity patterns show condition-specific modulation in their occurrence frequency, with Pattern 2, characterized by positive coherence between the VIS and FP networks and negative coherence between the DMN and VIS, FP, and SAL networks, exhibiting increased occurrence probability during movie watching compared to non-movie watching conditions; (2) participants show synchronization in their dynamic functional connectivity profiles during movie watching; (3) this synchronization is significantly associated with the narrative of the movie, and suspenseful moments are associated with higher synchronization; and (4) Pattern 2 contributed the most to the synchronization in suspenseful moments. Altogether these results suggest that processing of the same high-level information elicits similar functional connectivity dynamics that reflect and track changes in the properties of the processed information.

## Discussion

In the current study, we investigate how the processing of an engaging audio-visual stimulus alters ongoing brain activity and induces common functional connectivity dynamics across viewers. Until now, most studies investigating inter-subject synchronization during naturalistic stimuli viewing focused on local static activations^1–5,9,10^. In contrast, our study takes a global and dynamic perspective of neural activity and reveals that watching a plot-driven and captivating movie shapes the interaction of brain networks and synchronizes the network dynamics across-individuals. Importantly, we also demonstrate that the neural synchronization across participants is not stable but rather fluctuates over time. Indeed, the dynamics of the network interactions vary with the suspense of the scenes, resulting in a higher inter-subject synchronization when participants are immersed in the movie. These results were further confirmed in a second clustering procedure using the Harvard–Oxford atlas, which provided a more comprehensive representation of the brain compared to the 42 regions of interest employed in the initial analysis, ensuring that our findings were not dependent on the ROI selection.

In light of these results, we propose that suspense ratings may be used as a proxy for the allocation of attention. Suspenseful scenes capture attention which enhances the processing of the scenes, resulting in a higher inter-subject synchronization during these scenes. Compatible with our results, several studies have found that increased suspense in the narrative induces higher activity in the ventral attention network^28,29^. Our results go beyond this finding and show that increased attention in turn, modulates the ongoing processing similarly across individuals.

The two patterns that showed condition-specific modulation present both positive and negative phase coherence between different brain regions that coordinate according to the ongoing task (Fig. 2B). For example, positive coherence between visual and FP networks and negative coherence between DMN and FP networks during movie watching suggest that while attention networks coordinate with sensory areas, they decouple from the regions that are implicated in internal thought generation^30,31^. These results are consistent with previous studies reporting a juxtaposition between DM and FP networks during movie watching^32^ and during switches between internal and external awareness^33^, and furthermore show that this decoupling occurs simultaneously with the coupling of sensory and attention networks. Interestingly, studies show that patterns which exhibit both positive and negative coherence between different brain regions are predominant in healthy populations and diminish in unconscious states^23–26^. Our results add to this literature by showing that, in healthy participants, these rich coherence patterns can be divided into similar yet distinct sub-patterns reflecting the cognitive processes implicated in the ongoing task.

We believe that providing a comprehensive description of the fMRI patterns modulated by the experimental conditions is essential to uncover the underlying mechanisms at play. For instance, by thoroughly characterizing the network interactions within the brain patterns associated with conscious states, and delving into the sub-patterns that reflect the cognitive processes involved in the ongoing task (Pattern 1 and Pattern 2), we can potentially glean valuable insights into the specific network interactions that contribute to the formation of conscious experiences and our comprehension of the external world.

One could ask why scrambled movie watching and resting-state induce similar connectivity patterns although the two conditions differ drastically, especially at the sensory level. This might be due to the fact that participants could not follow the plot in the scrambled movie condition and therefore, may instead, have directed their attention to their self-generated thoughts, as they did during resting-state scans^30^. Since the whole-brain patterns capture the global brain state and not region-specific activations, they are likely more sensitive to participants’ ongoing mental activity than detailed sensory-driven processes, and thus, pick up on the similarities between the mental states elicited by these apparently different conditions.

In summary, our study provides valuable insights into the impact of ecologically valid stimuli on brain activity and inter-subject synchronization. By adopting a global and dynamic approach, we demonstrate that watching a plot-driven and captivating movie shapes the interaction of brain networks, resulting in synchronized network dynamics across individuals. This synchronization effect has potential applications in probing conscious processing across different states, including sleep, anesthesia, and disorders of consciousness (DoC). For instance, investigating the extent to which a DoC patient’s mental processes synchronize with those of healthy controls could help assess their ability to process external information. Such measures have the potential to improve the diagnostic process, particularly for patients displaying cognitive-motor dissociation^34^, who are often misdiagnosed as being in a vegetative state due to a lack of behavioral responses^35^. Overall, our study highlights the importance of considering dynamic network interactions and the high-level stimulus characteristics when exploring inter-subject synchronization during naturalistic stimuli viewing and hold implications for the development of tools that aimed at assessing intact cognitive capacities across a range of behaviorally unresponsive populations.

## Method

### Participants and procedure

In this study, we used a previously published dataset^5^ in which 27 participants underwent functional MRI recordings. During the acquisition, 15 participants (18–40 years; 7 males) watched an 8-min black and white movie clip taken from a TV show entitled “Alfred Hitchcock Presents—Bang! You’re Dead”. The same participants also went through a resting-state scan. A second group of 12 participants (18–30 years; 4 males) watched the same movie but in a scrambled order. In this condition, the movie was cut into 1-s segments and shuffled, allowing participants to see all the scenes without understanding the plot of the movie. Please note that these participants only underwent the scrambled movie condition and did not undergo any resting state scans. To evaluate how suspense varied in the movie, a third group of 15 participants (19–29 years; 5 males) watched the movie clip outside of the scanner and were asked to rate how suspenseful every 2-s segment of the movie was using an 8-point scale. All participants were right-handed native English speakers without any neurological or psychiatric disorders. They provided written informed consent prior to the experiment and were remunerated for their participation. This study followed the principles prescribed by the declaration of Helsinki and was approved by the local ethics board of the Western University. Further information on the participants or experimental procedure can be found in Naci et al.^5^.

### MRI acquisition parameters

MRI data were acquired on a 3T Siemens Tim Trio System. T2*-weighted whole-brain images were recorded during resting-state (256 volumes), movie (246 volumes) and scrambled movie (238) watching with a gradient-echo EPI sequence (33 slices, slice thickness: 3 mm, interslice gap of 25%, TR/TE: 2000 ms/30 ms, voxel size: 3 × 3 × 3 mm, flip angle: 75°). A mirror box allowed participants to see the movie that was presented on a projection screen behind the scanner. Noise cancellation headphones (Sensimetrics, S14) were also used for sound delivery. An anatomical volume was also acquired using T1-weighted MPRAGE sequence in the same acquisition sessions (154 slices, matrix size: 240 × 256 × 192, TE: 4.25 ms, voxel size: 1 × 1 × 1 mm, flip angle: 9°).

### ROI selection

Our main analysis incorporates 42 regions of interest (ROIs) belonging to six distinct networks (visual, auditory, saliency, default mode, fronto-parietal, and motor). These ROIs were adopted from a previous study^25^, which utilizes phase-synchrony-based functional connectivity patterns to distinguish between conscious and unconscious participants. In our research, one of our objectives was to explore the functional significance of these brain patterns in the context of conscious experiences and an understanding of a narrative. Our rationale was straightforward: if these brain patterns indeed mirror high-level cognitive processes occurring during the integration of intricate stimuli from various sensory modalities, then they should exhibit changes when participants engage in movie-watching, synchronizing among individuals who collectively share a conscious experience. To achieve this, we utilized the same set of ROIs as delineated in this previous study^25^. For a comprehensive list of these ROIs along with their MNI coordinates, please refer to Table S1. To ensure that our results were not biased by our ROI selection and could generalize to other brain parcellations, we conducted a second clustering procedure using the Harvard–Oxford atlas. This atlas comprises 48 cortical regions (91 ROIs in total when considering both hemispheres). The comprehensive list of ROIs used in this procedure is available on the NeuroVault website (https://neurovault.org/collections/262/).

### fMRI preprocessing

Raw MRI data were preprocessed and denoised using CONN functional connectivity toolbox^36^ implemented in MATLAB (The MathWorks). The first 5 volumes were discarded to ensure stable magnetization. The preprocessing procedure included realignment, slice-time correction, outlier detection, segmentation, normalization into the MNI152 space (Montreal Neurological Institute), and spatial smoothing using a Gaussian kernel of 6-mm full width at half-maximum. For outlier correction, images with more than 0.3 mm framewise displacement in one of the z, y, z directions, more than 0.02 rad rotational displacement, or global mean intensity exceeding 3 standard deviations were included as nuisance regressors in the generalized linear model (GLM). White matter and cerebrospinal fluid masks were also included as nuisance parameters in the GLMs. Average time-series from 42 regions of interest were extracted after applying a 0.008–0.09 Hz band-pass filter to the signal. Regions of interest were defined as 10 mm-diameter spheres around the given MNI coordinates (Table S1). Note that current recommendations for denoising procedures in fMRI analysis advocate for a simultaneous approach involving both band-pass filtering and nuisance regression in a single step^37,38^. However, in our main analysis, we chose to employ a sequential approach, first regression and then filtering. This decision was made to maintain methodological consistency with previous research that assessed phase-synchrony-based functional connectivity patterns in conscious and unconscious humans^25^. By following the same approach as these earlier studies, we ensured that the patterns observed in their work and our study would remain directly comparable. On the other hand, during our second clustering procedure, we chose to employ simultaneous band-pass filtering and nuisance regression. Given that the goal of this second clustering was to serve as a validation step rather than a direct comparison with previous studies, we optimized our preprocessing strategy accordingly.

### Time-varying functional connectivity patterns

All computations were performed in MATLAB. Following the preprocessing, extracted ROI time-series were represented in the complex space using their analytic representation that composed by the original signal (real part) and the Hilbert transform of the signal (imaginary part). The instantaneous phase is computed as the inverse tangent of the ratio of the imaginary and real components and wrapped into the [− *π*, *π*] interval using the angle function implemented in MATLAB. This allowed us to have a time-series of instantaneous phases for each ROI. Note that to avoid edge artefacts, the first and last 9 time points have been discarded from the time series. Then, phase differences between each ROI pair (861 in total) were calculated for every fMRI volume using cosine similarity. Thus, the whole-brain connectivity configuration at each time point could be represented as an observation in a 861-dimensional space. We concatenated data from all sessions (42 in total: 15 resting-states, 15 movies and 12 scrambled movies) and applied k-means clustering in order to uncover ‘prototypical’ connectivity configurations that were recurrent in all experimental conditions. Connectivity configuration at each time point was then labeled with one of the 4 cluster centroids to which the configuration belonged. Finally, participants’ brain activity during the scans was expressed as a sequence of the 4 centroids.

### K-means clustering procedure

K-means clustering is an unsupervised classification method allowing to divide a dataset into distinct, non-overlapping clusters. We selected K-means over other clustering methods because it doesn't require assumptions about the data's shape and has been previously used in similar studies^23–25^. The number of clusters, denoted as ‘k’, is determined in advance. In our study, we conducted the clustering process five times, considering k values of 3, 4, 5, 6, and 7. Subsequently, we employed the Silhouette method with elbow criterion to identify the optimal number of clusters our analysis revealed that 4 clusters provided the optimal classification. The algorithm operates as follows: Initially, the algorithm randomly selects K data points from the dataset as the initial cluster centroids. Then, it computes the distance between each data point and each cluster centroid, assigning each data point to the nearest cluster. In our study, we utilized the Manhattan distance as the distance metric. The subsequent step involves updating the cluster centroids. It calculates the new cluster centroids by computing the average of all the data points within each cluster. Once again, it computes the distance between each data point and each cluster centroid, reassigning each data point to the nearest cluster, and updating the cluster centroids. This process iterates until convergence, which occurs when the cluster centroids no longer change. Importantly, the choice of initial centroids can impact the final clustering outcome. Therefore, we repeated the clustering procedure 1000 times with different initial clusters to select the best result, which corresponds to the configuration with the lowest total sum of distances.

### Inter-subject similarity index

We computed an Inter-subject Similarity Index (ISI) in order to assess the resemblance between the participants’ pattern sequences using the following formula

$$ISI(A,B) = \frac{{\sum\nolimits_{i = 1}^{k} {\left( {P\left( {A = i,\;B = i} \right) - P\left( {A = i} \right)P\left( {B = i} \right)} \right)} }}{{\sum\nolimits_{i = 1}^{k} {\left( {\left( {P\left( {A = i} \right) + P\left( {B = i} \right)} \right)/2 - P\left( {A = i} \right)P\left( {B = i} \right)} \right)} }}$$

where P(A = *i*) and P(B = *i*) are the occurrence probabilities of Pattern *i* (among total number of k patterns, in this study k = 4) in participants A and B respectively. P(A = *i*, B = *i*) corresponds to the joint probability of exhibiting Pattern *i* by participants A and B at a given time-point. This index measures the co-occurrence of brain patterns between two participants (A and B) while correcting for the overall occurrence probability of each connectivity pattern. Positive values of ISI indicate higher co-occurrence of patterns compared to chance (ISI = 0). The synchronization level of each participant is defined as their average ISI with all the other participants of their group.

### Suspense rating

15 participants watched the movie outside of the scanner. The movie was cut into 2 s segments. After viewing each segment, participants rated the suspense level of the segment on an 8-point scale ranging from not suspenseful at all to maximal suspense. A moving average of 7 ratings (14 s) was computed in order to bring out the changes in the movie plot and remove the rapid variations within the scenes. Average suspense ratings can be found in Fig. 4A.

### Instantaneous co-occurrence

We used entropy as a measure of instantaneous pattern co-occurrence between participants. Entropy H(X) of the connectivity patterns at a given time point is computed with:

$$H\left( X \right) = - \sum\limits_{i = 1}^{4} {P\left( {x_{i} } \right)} \log_{2} P\left( {x_{i} } \right)$$

where P(*x*_*i*_) is the occurrence probability of pattern *i* among the participants at a given moment. Since we have four distinct patterns, the maximum entropy is equal to 2, indicating a uniform distribution of the pattern probabilities (¼ of the participants exhibited each pattern). Conversely, lower entropy values indicate that majority of the participants had the same pattern at the given time point. This co-occurrence measure is not pattern specific: increased co-occurrence of any patterns would decrease the entropy values. In order to assess the relationship between the suspense variations and the instantaneous co-occurrence among participants, we took the moving average of 7 entropy values as with the suspense ratings.

### Temporal pattern consistency

Following a previously published method^8^, we assessed which patterns contributed the most to the inter-subject co-occurrence of the patterns. This method consists in counting the number of participants having a given pattern at least once in a time window and in determining the pattern that manifested the most. Here we used a sliding window of 7 consecutive time points to construct the time windows similarly to all our other analyses. The dominant patterns during movie watching and scrambled movie watching can be found in Fig. 4C in the center and bottom panels respectively. The percentage of consistency indicates the percentage of participants having the dominant pattern at a time point. In order to exclude consistency that might appear by chance given the natural occurrence probabilities of the patterns, we subtracted the occurrence percentage of a pattern during the movie and scrambled movie-watching scans from the percentage of consistency of this pattern at a given time-point in these conditions.

### Statistical analyses

Occurrence probabilities of the patterns and changes in the similarity index across conditions are assessed with linear models using lme4^39^, car^40^, and emmans^41^ packages in R^42^. Wilcoxon rank-sum tests on the suspense ratings and the temporal consistency values, as well as the Spearman’s correlation between the suspense values and the entropy were performed on MATLAB (The MathWorks). All tests are corrected for multiple comparisons using the Bonferroni procedure except for the sign-tests on Pattern coherence levels which were corrected using the Benjamini–Hochberg procedure. We made this choice because our brain patterns consisted of 42 by 42 matrices, resulting in a total of 861 (42*41/2) values when comparing coherence values to zero. When dealing with such a substantial number of comparisons, applying the Bonferroni correction would have led to an exceedingly stringent significance threshold, potentially increasing the likelihood of false negatives. To address this issue, we instead opted for the Benjamini–Hochberg procedure with a significance threshold of 0.001, which is more stringent than the conventional 0.05 threshold.

### Supplementary Information

Supplementary Information.

## Data Availability

All data that support the findings of the study as well as the custom analysis scripts can be found in OSF (https://osf.io/ewuyk/?view_only=f540b0a6521b4e4b8b00fdaf5671d079).
