# Supplementary material for: Processing of the same narrative stimuli elicits common functional connectivity dynamics between individuals
Source: Sci Rep. 2023 Dec 2;13:21260. doi: 10.1038/s41598-023-48656-7 (PMC10692174; doi:10.1038/s41598-023-48656-7)
Supplement: Supplementary file 1 — Supplementary Information. [file 41598_2023_48656_MOESM1_ESM.docx]

**SUPPLEMENTARY FIGURES**

**
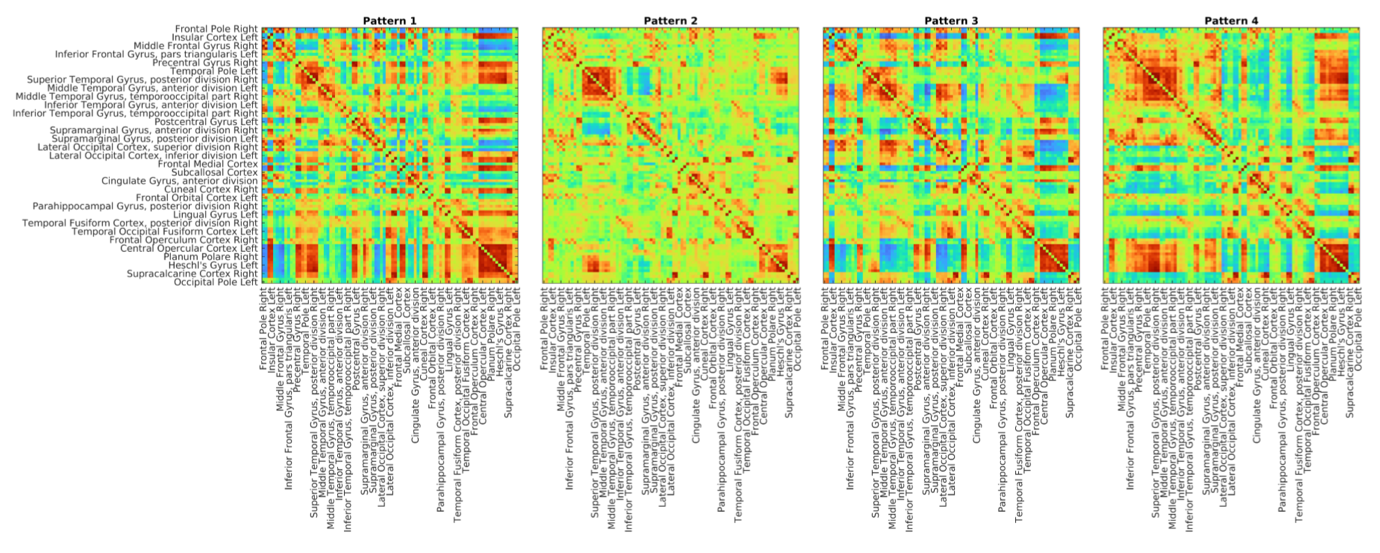
**

**Figure S1. The four cluster centroids obtained via k-means clustering using the Harvard-Oxford parcellation.** Each pattern is represented as a 91 by 91 coherence matrix. Please note that due to visualization constraints, not all ROI names are displayed on the x and y axes. The complete list of ROIs used in this procedure is available on the NeuroVault website (https://neurovault.org/collections/262/).

**
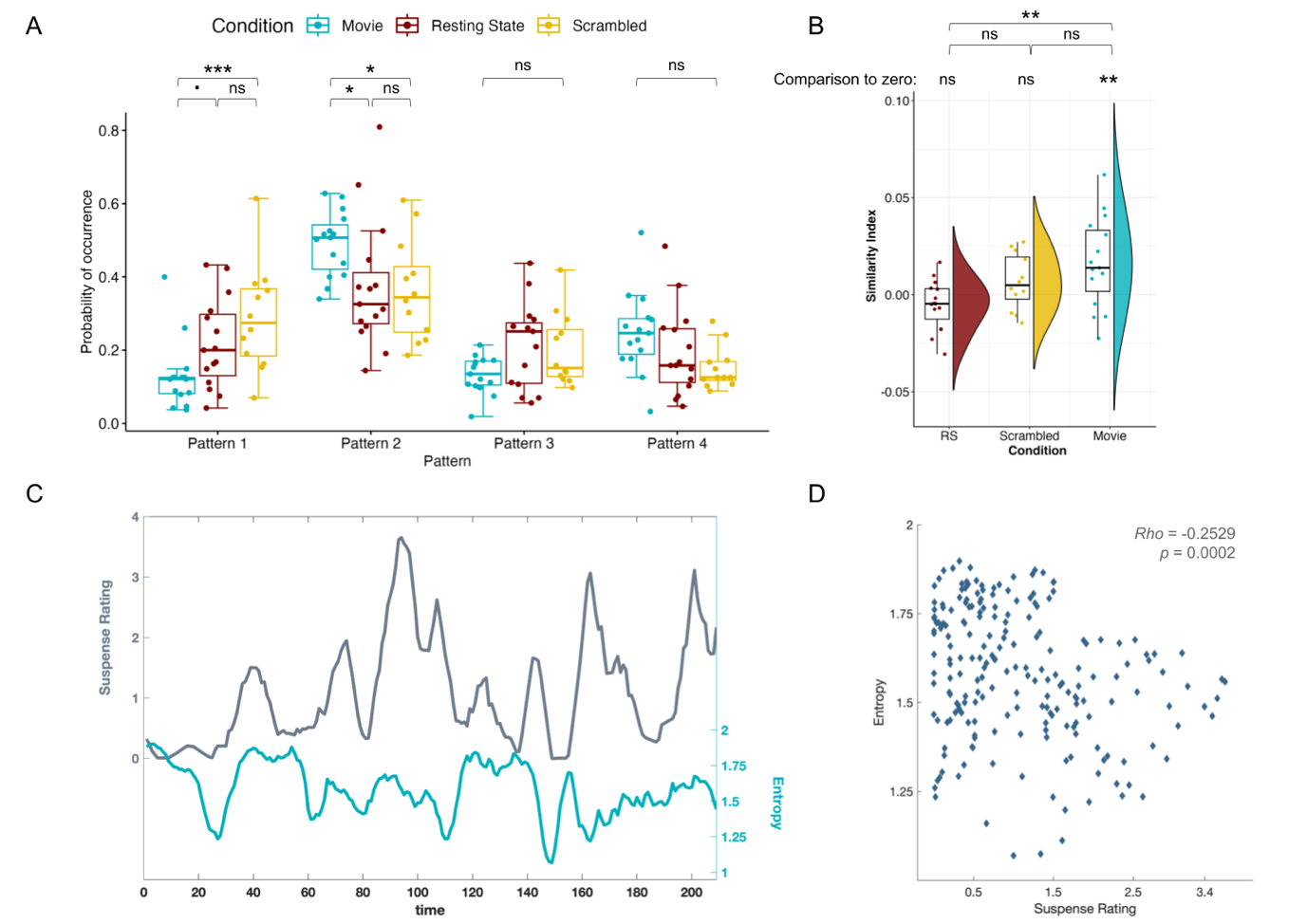
**

**Figure S2. Results from the clustering procedure using the Harvard-Oxford atlas.** (A) Occurrence probability of the 4 patterns during movie watching (blue), rest (brown) and scrambled movie watching (yellow). Patterns 3 and 4 exhibited similar occurrence probabilities across the three conditions. However, Patterns 1 and 2 displayed condition-specific modulation: Pattern 2 had an increased occurrence during movie watching, while Pattern 1 was more frequent in the scrambled movie watching and resting-state conditions. Stars indicate statistical significance, while the dot represents a tendency (*p* = 0.057) after correction for multiple comparisons using the Bonferroni procedure. (B) Movie watching induces inter-subject synchronization Average inter-subject similarity index (ISI) of each participant in resting-state (RS), scrambled movie, and movie watching conditions. Similarity indices were not different than zero in the resting state (*V* = 33, *p* = 0.14) and scrambled-movie watching (*V* = 57, *p* = 0.18) conditions, indicating a chance-level co-occurrence of patterns among participants throughout the whole duration. On the other hand, participants in the movie watching condition showed higher similarity index values (*V* = 100. *p* = 0.021), indicating increased co-occurrence of the patterns compared to chance. The ISI values were also statistically different between resting state and movie watching conditions (t(39) = -3.389, *p* = 0.0049). Each dot represents a participant. (C) Scenes with high suspense induce inter-subject synchronization. Suspenseful scenes increase pattern co-occurrence during movie watching. The dark gray line shows the variations in the average suspense rating. Entropy (light blue line) of the pattern distribution among participants at each time point is used as an instantaneous co-occurrence measure. Lower entropy values indicate higher co-occurrence. Note the negative relationship between the suspense rating and the entropy values: scenes with higher suspense ratings are followed by a decrease in entropy and thus an increased co-occurrence of the patterns among participants. (D) Scatter plot of the average suspense rating and the subsequent entropy values (6 seconds after the suspense rating). We found a significant negative correlation between the two measures (*rho* = -0.2529; *p* = 0.0002).

**
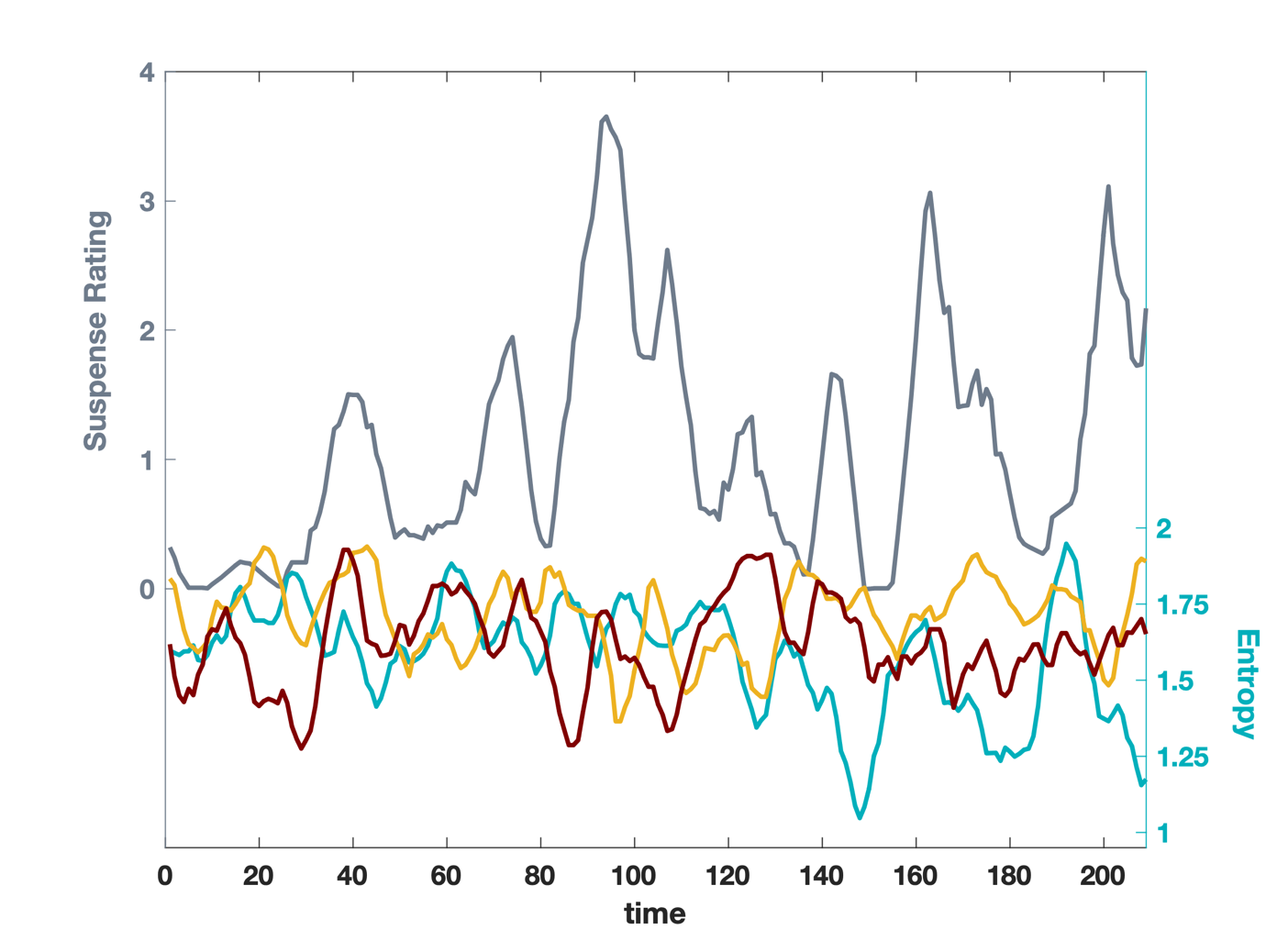
Figure S3. The relationship between the suspense rating and the entropy values in the movie (blue lines), resting-state (red lines) and scrambled movie (yellow lines) conditions.** Moments with increased suspense was followed by a decrease in the entropy values in the movie watching condition. This negative relationship was not found in the resting-state and the scrambled movie watching conditions.

**Table S1. 42 regions of interest used in the inter-areal coherence analyses.** The regions were defined as 10mm-diameter spheres around the given x,y,z coordinates.

| **Region of Interest (ROI)** | **Seed MNI coordinates [x, y, z]** |
| --- | --- |
| **Auditory Network (AUD)** | |
| Anterior cingulate cortex | [6, -7, 43] |
| Precentral gyrus [left] [right] | [-53, -6, 7] [58, -6, 11] |
| Superior transverse temporal gyrus [left] [right] | [-44, -6, 11] [44, -6, 11] |
| **Default Mode Network (DMN)** | |
| Inferior temporal cortex [left] [right] | [-61, -24,-9] [58, -24, -9] |
| Lateral parietal cortex [left] [right] | [-46, -66, 30] [49, -63, 33] |
| Medial prefrontal cortex | [-1, 54, 27] |
| Posterior cingulate cortex | [0, -52, 27] |
| **Fronto Parietal Network (FP)** | |
| Angular gyrus [left] [right] | [-31, -59, 42] [30, -61, 39] |
| Midcingulate cortex | [0, -29, 30] |
| Premotor cortex left [left] [right] | [-41, 3, 36] [41, 3, 36 ] |
| Inferior parietal lobule [left] [right] | [-51, -51, 36] [51, -47, 42] |
| Dorsolateral prefrontal cortex [left] [right] | [-43, 22, 34] [43, 22, 34] |
| **Motor Network (MOT)** | |
| Supplementary motor area | [0, -21, 48] |
| Primary motor cortex [left] [right] | [-39, -26, 51] [38, -26, 48] |
| **Saliency Network (SAL)** | |
| Dorsolateral prefrontal cortex [left] [right] | [-38, 52, 10] [30, 48, 22] |
| Ventrolateral prefrontal cortex | [42, 46, 0] |
| Parietal operculum [left] [right] | [-60, -40, 40] [58, -40, 30] |
| Supplementary motor area [left] [right] | [-5, 14, 48] [5, 14, 48] |
| Dorsal anterior cingulate | [-6, 18, 30] |
| Paracingulate cortex | [0, 44, 28] |
| Temporal pole [left] [right] | [-50, 14, -14] [51, 16, -19] |
| Orbital frontoinsula [left] [right] | [-40, 18, -12] [42, 10, -12] |
| **Visual Network (VIS)** | |
| Associative visual cortex [left] [right] | [30, -89, 20] [-30, -89, 20] |
| Secondary visual cortex [left] [right] | [-6, -78, -3] [6, -78, -3] |
| Primary visual cortex [left] [right] | [-13, -85. 6] [8, -82, 6] |
